# Clinically relevant results of reverse total shoulder arthroplasty for patients younger than 65 years compared to the older patients

**DOI:** 10.1186/s42836-021-00086-4

**Published:** 2021-09-01

**Authors:** Klaus Hanisch, Michael Boelstoft Holte, Inge Hvass, Niels Wedderkopp

**Affiliations:** 1 The Orthopedic Department, University hospital of South West Denmark, Esbjerg, Denmark; 2 Department of Regional Health Research, Faculty of Health Sciences, University of Southern Denmark, Odense, Denmark

**Keywords:** Reverse total shoulder arthroplasty, Young patients, Western Ontario osteoarthritis index

## Abstract

**Background:**

Reverse total shoulder arthroplasty was originally designed for older patients with rotator cuff arthropathy and produces good results. The main objective of this retrospective study was to compare the patients younger than 65 years *vs*. the older patients in terms of the complications of reverse total shoulder arthroplasty and the functional recovery.

**Methods:**

From January 2014 to January 2020, 566 patients who underwent the reverse total shoulder arthroplasty were divided into two groups (group A, ≥ 65 years, *n* = 506; group B, < 65 years, *n* = 60). The patients reported the quality of life using the patient-reported Western Ontario Osteoarthritis of the Shoulder index. The Constant score was obtained preoperatively and 3 months postoperatively. The complications and reoperations were compared. Statistical significance was set at *P* < 0.05.

**Results:**

Clinically relevant improvements were found in group A and B. There was a multivariate statistically-significant but not clinically relevant difference in the change over time between group A and B. The mean 12-month Western Ontario Osteoarthritis of the Shoulder indexes were 58 in group B and 71 in group A. The mean Constant scores were 44 in group B *vs*. 43 in group A. Compared to group A, group B had a non-significant odds ratio of 1.9, which did not reach the clinically relevant Western Ontario Osteoarthritis of the Shoulder index of group A.

**Conclusion:**

In patients younger than 65 years of age, RTSA seems to be a safe procedure in short term follow-up. After 1 year, we found no increased risk of complications, revision, or inferior outcomes compared to patients older than 65 years of age. Consequently, after one-year, RTSA provided clinically relevant improvements in the patients’ quality of life and shoulder strength regardless of age.

## Introduction

Combined rotator-cuff insufficiency and glenohumeral osteoarthritis is a challenging clinical entity for the orthopaedic surgeons. Reverse total shoulder arthroplasty (RTSA) has provided excellent outcomes for older patients afflicted with the painful and functionally-limited shoulders. However, few studies have evaluated the outcomes of RTSA used in younger patients.

The RTSA was initially designed for older patients with rotator-cuff insufficiency and glenohumeral osteoarthritis, but the indications are expanding. Humeral fractures, fracture sequelae, and shoulder osteoarthritis, especially in combination with glenoid dysplasia, rheumatoid arthritis, or caput necrosis, may be good indications for RTSA. Thus, more and more younger patients aged < 65 years become candidates. However, the long-term effect and complications of RTSA used in younger patients are still unclear. The Grammont RTSA has been developed for this purpose. The Grammont prosthesis is effective for relieving pain and restoring active movement in patients with glenohumeral osteoarthritis and rotator-cuff insufficiency [1]. Compared to the early arthroplasties, such as total shoulder arthroplasty and shoulder hemi-arthroplasty, the RTSA also produces good results in rotator cuff arthropathy and osteoarthritis. However, as a new technique, the postoperative complications and side effects are not totally clear, including scapular notching, arm extension lag, resorption of tuberculum major, *etc*. [2, 3].

In a recently published review (covering 245 participants in 6 studies) by Vancolen *et al*. [4], the pooled mean complication rate was as high as 18%, because the revision rate was 36% due to failed arthroplasties and older patients were included in the studies. Ek *et al*. [5] reported their results based on the operations performed between 1997 and 2006. In their study, the Constant scores increased from a mean score of 34 to 74, and the mean subjective shoulder values improved from 23 to 66. The complication rate was as high as 38% (15 of 35 patients), because the follow-up period lasted 93 months (range, 60 to 171 months).

The aim of this study was to compare the patients younger than 65 years *vs*. the older patients in terms of the complications of RTSA and functional recovery.

## Material and methods

From January 2014 to January 2020, 566 consecutive patients who underwent RTSA at the Orthopedic Department of the University Hospital of South West Denmark were included in the study. All patient data were entered into the shoulder registry database before surgery. The patients were divided into two groups (group A, ≥ 65 years; group B, < 65 years). We used cemented and uncemented Delta Xtend prostheses (Depuy, Orthopaedics, Inc., IN, USA), and uncemented Univers Reverse Shoulder Arthroplasty System (Arthrex, Naples, FL, USA) in this study. The patients reported the quality of life using the patient-reported Western Ontario Osteoarthritis of the Shoulder (WOOS) index, preoperatively, and 3 months and 1 year after surgery. The Constant score was obtained preoperatively and 3 months postoperatively. The complications treated and reoperations were registered and entered into the database.

### Statistical analysis

The continuous data with standard normal distribution were expressed as mean and standard deviation. The continuous variables with asymmetrical distribution were presented as median and interquartile range. The normality was checked visually by using standardized normal probability plots. Categorical data were reported as numbers and proportions. Bivariate comparisons were made between the two groups. Both the bivariate and the multivariate analyses were performed using mixed linear regression, taking into account the repeated measurements and the possible clustering of result on surgeon. Logistic regression was used to estimate the probability of not reaching a clinically relevant result, as this analysis was performed with the dichotomized 12-month follow-up WOOS and adjustment for the baseline WOOS. We used robust standard errors with the “cluster option” of STATA to take into account the possible clustering of results on surgeons. The WOOS was dichotomized according to the Danish validation of WOOS, where a score greater than 50% (950) was considered clinical relevant and “good” [6].

The multivariate analyses were adjusted for sex and type of diagnoses. The diagnoses were categorized into (1) fracture and failures after other primary treatments, (2) osteoarthritis with cuff arthropathy, and (3) others. The interaction terms between the time points, baselines, 3 and 12 months, and the dichotomized age variable (< and ≥ 65 years) were entered to estimate the difference between the groups over time in the mixed linear regression. We further used the interaction term between the diagnoses and dichotomized age over time to assess the effect of diagnoses in the groups. Marginal effects were calculated and used to determine the difference between the groups in WOOS scores and Constant score at the follow-up time points of 3 and 12 months. In addition, the WOOS score 12 months after operation was dichotomized, according to the validated Danish version of WOOS [6].

The normality of the residuals was checked for establishing the analyses. The results were graphically illustrated using the predicted outcome and probability of WOOS above 950 as validated or 50% of maximum score by the groups [6]. Longitudinal analyses were performed, and all data were used at all time points to increase the statistical power of the analyses. All analyses were performed using STATA MP 16.1.

## Results

In a total of 566 patients, 366 were female and 200 were male patients. Among them, 335 female and 171 male patients were allocated to group A (≥ 65 years, *n* = 506), with 372 patients having 3-month and 196 having 12-month follow-up data. Group B (< 65 years, *n* = 60) included 31 female patients and 29 male patients. Of them, 54 patients had 3-month and 30 had 12-month follow-up data. The demographics of the groups are shown in Table 1. All scores were normally distributed except for the WOOS at 12-month follow-up. Thus, the data showed the 25th and 75th percentiles instead of standard deviation. There was no difference in distribution of diagnoses across ages (*P* = 0.290) or between the groups (*P* = 0.216) (Table 1).

**Table 1:** Demographics of the patients receiving a reverse shoulder arthroplasty from 2014 to 2020

				Preop score		Postop score		
	Sex	*n*	Mean age (yr)	WOOS (SD)	Constant (SD)	WOOS 3 months (SD)	WOOS 12 months (25th;75th pctile)	Constant 3 months (SD)
Group B (< 65 years)								
Delta Xtend	Female	31	60	22.2 (11.0)	18.9 (9.0)	53.0 (23.7)	59 (41; 68)	39.7 (18.7)
Cemented:	Male	22	57	21.9 (13.7)	23.8 (11.5)	56.7 (31.2)	82 (47; 89)	50.3 (25.3)
Delta Xtend	Female	2	58	35.0 (17.5)	19 (8.5)	74.2 (29.0)	35 (8; 63)	49.5 (24.7)
Uncemented:	Male	3	62	16.1 (7.9)	23 (1.4)	83.1 (10.4)	25 (11; 65)	56 (21.2)
Univers Reverse:	Female	4	62	22.4 (14.7)	19.8 (9.2)	46.8 (36.4)	94 (94; 94)	34.7 (21.5)
Univers Reverse:	Male	6	61	15.3 (6.3)	14.3 (7.9)	39.6 (27.9)	25 (11; 65)	33 (17.0)
Group A (≥ 65 years)								
Delta Xtend	Female	313	77	24.4 (15.8)	20.2 (10.3)	58.8 (24.5)	75 (49; 91)	39.0 (16.9)
Cemented:	Male	139	76	32.0 (17.9)	22.7 (11.0)	65.4 (24.5)	86 (61; 94)	46.8 (20.7)
Delta Xtend	Female	8	79	25.1 (13.0)	25.3 (5.4)	64.9 (15.6)	82 (82; 82)	39.8 (16.9)
Uncemented:	Male	21	74	34.1 (18.3)	23.4 (8.7)	70.0 (22.2)	71 (55; 86)	50 (18.6)
Univers Reverse:	Female	8	75	26.7 (11.7)	18.5 (5.1)	56.7 (9.8)	70 (54; 91)	36.4 (17.6)
Univers Reverse:	Male	9	72	37.2 (8.4)	24.2 (7.6)	56.0 (26.9)	71 (71; 71)	39.0 (15.5)

*SD* standard deviation, *Pctile* percentile

**Fig. 1:**
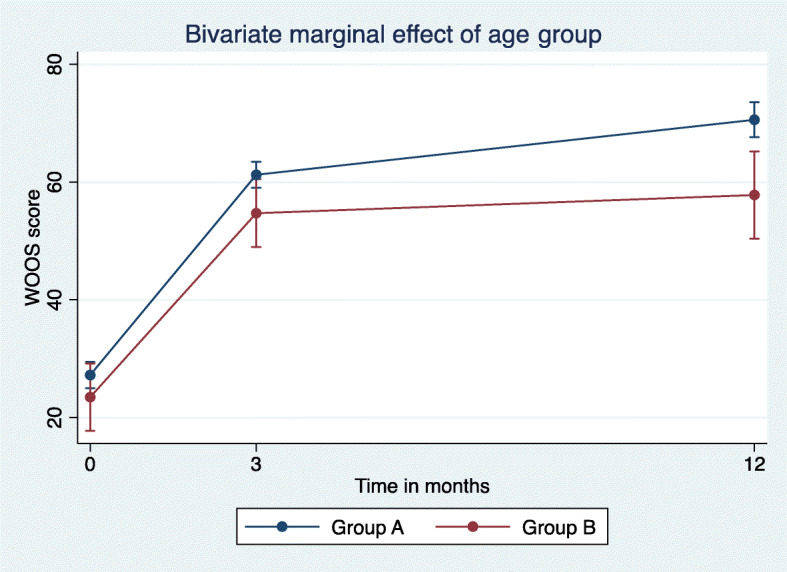
Estimated bivariate marginal change over time in WOOS with 95% CI significantly changed in both groups, and the 65 or older group had a significantly greater improvement, but the differences in score and improvement between groups were not clinically relevant

**Fig. 2:**
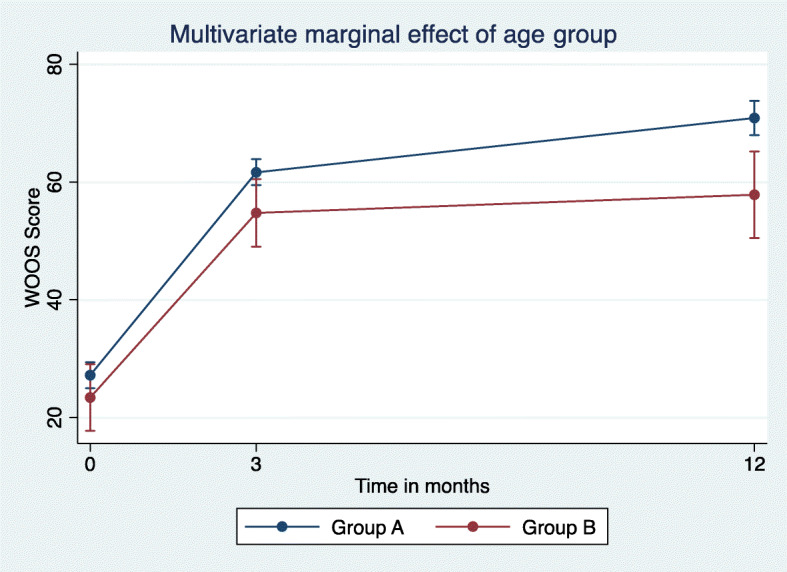
Estimated multivariate marginal change over time in WOOS with 95% CI significantly changed in both groups, and the older than 65 group had a significantly greater improvement

**Fig. 3:**
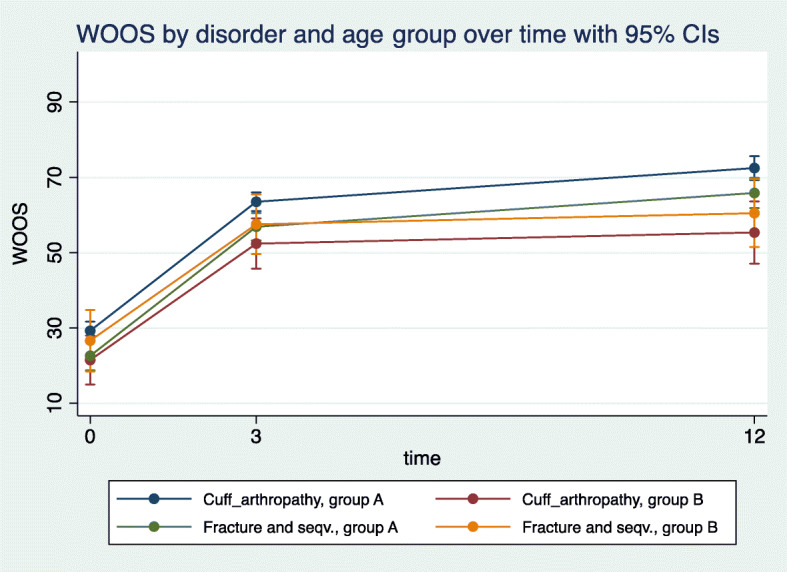
Estimated multivariate marginal change over time in WOOS with 95% CI was statistically significant in both groups. Comparing the WOOS over time of fracture and fracture sequalae diagnoses with cuff arthropathy, a statistically significant difference between the two groups was found

**Fig. 4:**
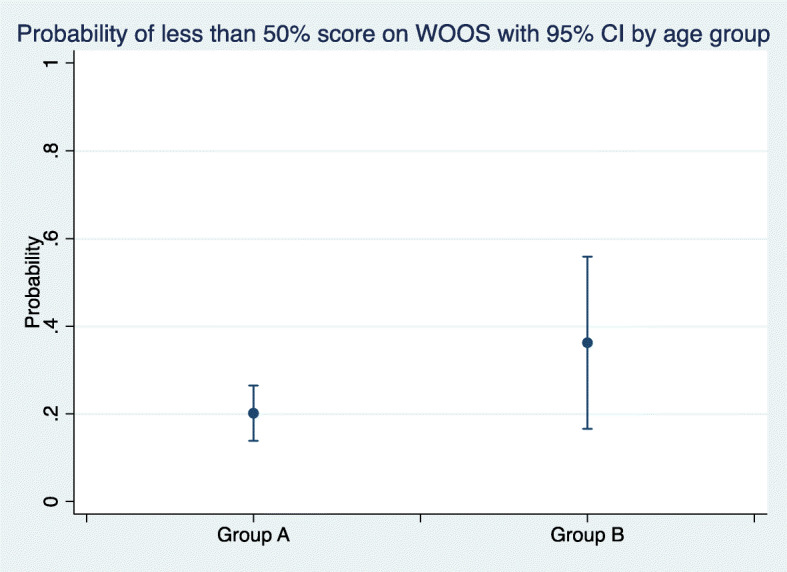
The probability of not reaching a clinically relevant results in a score higher than 50%, according to the Danish version of WOOS. There was no significant difference between the two age groups

WOOS and Constant scores improved significantly as shown by both the bivariate and multivariate analysis of marginal effects in the two groups. Both the bivariate and multivariate analyses showed clinically relevant improvements. There was a statistically significant but not clinically relevant difference in the change over time (Figs. 1 and 2). However, this difference disappeared when the interaction between the disorder and groups over time was introduced. The only difference appeared between the diagnoses of fracture and fracture sequelae *vs*. cuff arthropathy (Fig. 3). In group A, there was in significantly increased odds ratio (1.9) (95% CI 0.8; 9.8) with a WOOS score less than 950/50% at 3- and 12-month follow-ups. And 11 patients had a low WOOS. In group B, 46 patients had a low WOOS. The probability of not reaching a clinically relevant level of WOOS score of reaching a clinically relevant improvement was 22% in group A and 36% in group B (Fig. 4).

Postoperative complications occurred in 43 patients (Table 2). Among them, 7 (10%) patients were in group A and 26 patients underwent an arthroplasty due to proximal humeral fractures. For the patients who were treated for proximal humerus fractures, the multivariate odds ratio was 6.9 (*P* < 0.0001) and the WOOS was smaller than 950/50%, compared to the patients who had no fractures. The mean WOOS at 12 months was 56 and 68% for the patients with and without fractures, respectively. All infections were treated successfully by using antibiotics, and only one patient underwent reoperation and ended up with a “shoulder-girdlestone”. No patient received a revision arthroplasty. In group B, odds were not elevated regarding the complications. The multivariate odds ratio was 1.1 (95% CI, 0.2; 5.1) (*P* = 0.90).

**Table 2:** Postoperative complications

	Group A	Group B
	(≥65 years; *n* = 36; 90%)	(< 65 years; *n* = 7; 10%)
Infection	10	3
Wound rupture	1	0
Suture granuloma	1	0
Dislocation	21	2
Fracture	5	1
Axillary nerve palsy	1	1
Reoperation	1	0

## Discussion

Our results showed that the RTSA is a good and safe treatment for the patients younger than 65 years. The treatment was associated with high WOOS and Constant scores, without increased risk for complications.

Our results were generally in agreement with the systematic reviews conducted by Chelli *et al*. [7, 8] and Bedeir *et al*. [7, 8]. When the indications for RTSA are expanding to patients younger than 65 years, the early concern is that young age may be associated with higher complication and revision rates [4]. In our series, reoperation was associated with the proximal humeral fractures. However, the incidence of fracture is relatively low in cuff arthropathy due to osteoarthritis [9].

In a review by Vancolen *et al*. [4], the pooled mean complication rate of the RTSA was 18%, with the major and minor complication rates being 13 and 5%, respectively. However, it was likely that the rates were over-estimated due to a large number of revision, older implants, and the heterogeneity of patients and indications. Our study showed that the complication rate was 10%, because the data were based on the patients younger than 65 years of age. Scapular notching was first reported in the Grammont prostheses [10]. This complication did not occur in our series because the prostheses were not used in our series. When looking at the diagnoses, we found that patients with fractures and fracture sequelae had a higher odds rate of complications. It was in accordance with the earlier findings obtained in shoulder arthroplasty, including RTSA [11, 12]. No revision was performed in our series, but there was an increased odds rate of the complications in patients who had fracture and fracture sequelae.

Compared to the early studies [4, 5, 7, 8], the strengths of our study included a relative large number of patients, a comparison between two groups, the use of patient-reported outcomes, the use of longitudinal analyses, and the mixed regression techniques that improve the statistical power. This study had some limitations. First, it is not a randomized or longitudinal study, thus the type of causal pathways could not be determined. The operations were performed in a single hospital and by 3 senior surgeons, which might differ from the early studies conducted in the multiple centers and the surgical skills also improve with time [4, 5]. The choice of cemented or uncemented RTSA depends on the surgeon’s preference and surgical plans, which increased the variability of the results. Our follow-up period was relatively short for some complications to show up.

## Conclusion

In patients younger than 65 years of age, RTSA seems to be a safe procedure in short-term follow-up. After 1 year we found no increased risk of complications, revision, or inferior outcomes compared to patients older than 65 years of age. Consequently, after one-year, RTSA provided clinically relevant improvements in the quality of life and shoulder strength regardless of age.

## Data Availability

The data that support the findings of this study are available from The Danish Clinical Quality Program – National Clinical Registries (RKKP) (https://www.rkkp.dk/in-english/) but restrictions apply to the availability of these data, which were used under license for the current study, and so are not publicly available. Data are however available from the authors upon reasonable request and with permission of The Danish Clinical Quality Program – National Clinical Registries (RKKP) (https://www.rkkp.dk/in-english/).

## Abbreviations

RTSA: Reverse total shoulder arthroplasty
WOOS: The Western Ontario Osteoarthritis of the Shoulder index
SD: Standard deviation
